# Multimodal Remote Research on Social Anxiety Using a New Teleconferencing Paradigm

**DOI:** 10.1007/s10608-023-10371-y

**Published:** 2023-04-13

**Authors:** Mikael Rubin, Eli S. Gebhardt, Luna Malloy, Michael J. Telch

**Affiliations:** 1 Department of Psychology, The University of Texas at Austin, Austin, TX USA; 2 Department of Psychology, Palo Alto University, Palo Alto, CA USA

**Keywords:** Social Anxiety, Internet-Based Research, Social Stressor Challenge, Eye Tracking, Audio Vocal Indicators

## Abstract

**Background:**

Social anxiety is a prevalent mental health concern. Models of social anxiety incorporate multifaceted components from cognitive appraisals to attention as factors maintaining the disorder. Multimodal research investigating multiple facets of social anxiety simultaneously offers an important avenue to advance our understanding of the disorder.

**Methods:**

The current study tested a novel, internet-based simulated teleconferencing interaction social stressor challenge and included the collection of self-report, eye-tracking, and auditory vocal data during the challenge. Participants (N = 262) completed two interactions. The pre-recorded male and female audience members (assigned to display interest or uninterest) discussed a topic and then prompted the participant to speak on that topic.

**Results:**

Fidelity indices revealed that most participants rated the interactions with the simulated audience as realistic; reported heightened subjective distress during the simulated teleconferencing interactions; and correctly rated audience members’ level of interest. As predicted, social anxiety predicted participants’ subjective distress during the simulated teleconferencing interactions. Findings from audio vocal and eye tracking analyses largely corresponded to prior research – indicating that social anxiety influences audio vocal responses and patterns of attention during social stressors.

**Conclusions:**

Taken together, these findings suggest that the simulated teleconferencing interaction framework introduced here offers a potentially useful approach for the remote investigation of mechanisms underpinning social anxiety.

**Supplementary Information:**

The online version contains supplementary material available at 10.1007/s10608-023-10371-y.

Social anxiety disorder (SAD) is a common mental health concern (Kessler et al., 2005) that has a significant impact on quality of life (Rapaport et al., 2005). Models of social anxiety suggest several potential mechanisms that may maintain the disorder (Wong & Rapee, 2016). However, elucidating these factors remains an ongoing effort. Public speaking challenges have been effectively used to identify factors implicated in the pathogenesis of SAD (Croft et al., 2004) as well as its treatment (Hindo & González-Prendes, 2011; Niles et al., 2015). While the most common format for public speaking challenges has been in person, with a live audience, more recent research has also used a pre-recorded audience presented on a computer screen (Chen et al., 2015) or in virtual reality (Felnhofer et al., 2014). When conducted in conjunction with eye tracking (Reichenberger et al., 2020; Rubin et al., 2020), there is also opportunity to study attentional processes during the challenge itself. Findings from this line of research have suggested that during public speaking there may be avoidance of socially threatening audience members (although one study showed hypervigilance to audience members; Lin et al., 2016). Additionally, research investigating vocal acoustic indicators through audio capture during public speaking challenges (Weeks et al., 2012), has shown associations between social anxiety and vocal pitch (the auditory impression resulting from fundamental frequency – the opening and closing of the vocal folds during speech) as well as vocal intensity (Galili et al., 2013). Taken together these results highlight the potential for public speaking challenges to be used as a paradigm for elucidating mechanisms underpinning social anxiety.

## Remote Research and Social Anxiety

During the COVID-19 pandemic, the assessment and treatment of social anxiety shifted to being conducted remotely, via teleconferencing platforms. In seeking to address this need, researchers have investigated ways to conduct research on social anxiety remotely – for instance with a modified version of the Trier Social Stress Test (Huneke et al., 2021). Yet, the teleconferencing modality itself has yet to be tested as a distinct social stressor, despite data suggesting that individuals with social anxiety are more likely to avoid digital interactions (Arad et al., 2021). Moreover, there is preliminary evidence that teleconferencing for the treatment of social anxiety can be effective (Nauphal et al., 2021; Yuen et al., 2019). However, organizing a public speaking challenge using a real audience is logistically challenging (even with teleconferencing) and may introduce unwanted confounding variables. There is a wealth of prior research using pre-recorded audiences for public speaking challenges (typically in virtual reality, e.g., Reeves et al., 2021), indicating their effectiveness in eliciting social-evaluative threat - even when the participants know that the audience is pre-recorded (Rubin et al., 2020; Rubin et al., 2022). A remote teleconferencing paradigm for studying social evaluative threat would offer a highly scalable and flexible way to study social evaluative threat. Moreover, the development of software that can collect voice recording and eye tracking via the internet provides opportunities to generate large multimodal datasets that can yield new insights into factors underpinning the maintenance of social anxiety.

## The Current Study

Here we report on a novel simulated teleconferencing interaction framework for conducting research on social anxiety remotely. The simulated teleconferencing interaction consisted of four individuals discussing a topic and then prompting the participant to discuss their thoughts on the topic (e.g., their favorite book). Remote data collection also facilitated the incorporation of a range of indicators (including self-report, vocal acoustic, and eye tracking) to provide a robust assessment of social anxiety in response to an ecologically relevant context. In the current paper we focus first on showing that the simulated teleconferencing interactions were realistic and effective at eliciting fear. Second, we investigated the role of social anxiety symptoms in predicting self-reported distress, audio vocal indicators, and gaze behavior. We hypothesized that (1) greater endorsement of subjective fear would be associated with greater symptoms of social anxiety; (2) that greater social anxiety would be associated with greater pitch, decreased loudness, and shorter speaking duration and (3) greater social anxiety would be associated with avoidance of socially threatening (uninterested) audience members compared to interested audience members. Additionally, we explored the role of interaction order (first and second) as a potential moderator.

## Method

### Participants

266 undergraduate students who were enrolled in an introductory psychology course completed the study online for course credit as part of the baseline assessment for a clinical trial testing an internet-based intervention for social anxiety. Recruitment primarily took place through the undergraduate research platform, SONA. The study was approved by the University of Texas at Austin Institutional Review Board.

### Procedures

Participants completed the study using the online survey platforms Qualtrics (Qualtrics, Provo, UT) and Gorilla Experiment Builder (Anwyl-Irvine et al., 2020). Participants first provided informed consent – there was no deception involved in the study, so participants were informed that the interactions were pre-recorded. Participants then completed demographic questions as well as several questionnaires related to social anxiety on Qualtrics. Participants were then redirected to Gorilla where they completed two simulated teleconferencing interactions. Participants were told that they would be interacting with a pre-recorded audience who have been instructed to speak among themselves and then to ask the participant their opinion about the topic of the conversation. Participants viewed a brief introductory video (a recording of one of the audience members) telling the participant what the topic of the conversation would be and that when they were asked to respond they should continue until the screen went blank (indicating a shift in topic). During the conversation (within the first minute or so), one audience member addressed the participant by asking “what do *you* think about [this] topic?” Before and after each interaction, participants were asked to complete visual analogue scales indexing level of subjective distress on a 0-100 Likert scale. Before each interaction there was a brief calibration procedure for the web camera eye tracking. Participants were asked a question by a male and female audience member in the two separate counter-balanced simulated teleconferencing interactions. After completing both interactions, participants were directed to complete questions regarding their perceptions of the audience members and of the task.

## Measures

### Liebowitz Social Anxiety Scale Self Report Version (LSAS-SR)

The LSAS self-report scale (Liebowitz, 1987) is a 48-item measure of fear and avoidance concerning social interactions and performance situations (e.g. telephoning in public, talking to people in authority). Participants rate each item on a 0–3 Likert scale for Fear or Anxiety (0 = “none”, 3= “severe”) and Avoidance (0= “never (0%) to 3 = “usually (67–100%) with a score ranging from 0-144. The LSAS-SR showed evidence of high internal reliability 0.96 in the current sample.

### Personal Report of Communication Apprehension (PRCA)

The PRCA (McCroskey et al., 1985) is a 24-item instrument that is designed to assess anxiety related to speaking in a variety of situation. Participants rate their agreement with statements such as “I am afraid to express myself at meetings” on a 1–5 scale (1 = strongly disagree, 5 = strongly agree), with a score ranging from 24 to 120. The PRCA showed evidence of high internal reliability 0.95 in the current sample.

### Subjective Units of Distress Scale (SUDS)

SUDS were evaluated using a (0-100) visual analog scale to assess the degree of fear associated with completing the next trial (anticipated fear); following the simulated teleconferencing interaction SUDS were used to evaluate the greatest degree of threat experienced during (peak fear) and the degree of fear experienced currently (end fear).

### Audience Interest

Participants were shown a grid of the audience member faces from the simulated teleconferencing interaction with a key to identify each one by letter (A-D) and asked to rate the audience members interest from 0 (not at all interested) to 10 (extremely interested).

### Emotional Reaction to Audience Member

Participants were shown a grid of the audience member faces from the simulated teleconferencing interaction with a key to identify each one by letter (A-D) and asked to rate their emotional reaction to that audience member from 0 (extremely negative) to 10 (extremely positive).

### Demographics

Participants were asked to provide demographic information including sex, gender, age, race, and ethnicity on the internet prescreen (Table 1).

**Table 1:** Participant Demographics (N = 262)

Variable	M (SD)
Age	18.98 (1.17)
LSAS	63.27 (26.83)
PRCA	56.07 (17.42)
Subjective Units of Distress Scale (SUDS)	
Anticipated Fear	44.03 (28.53)
Peak Fear	39.54 (29.72)
End Fear	23.35 (27.91)
	N (%)
Biological sex (Female)	201 (76.7)
Gender identity	
Cisgender	238 (91.0)
Genderfluid	1 (0.3)
Non-binary	6 (2.3)
Transgender	1 (0.3)
Declined to report	16 (6.1)
Ethnicity (Hispanic/Latinx)	76 (29.0)
Race	
American Indian or Alaska Native	0 (0)
Asian	74 (28.3)
Black or African American	14 (5.3)
Hispanic or Latino/Latina/Latinx	42 (16.0)
Native Hawaiian or Other Pacific Islander	0 (0)
White	97 (37.1)
More than one Race	34 (13.0)
Declined to report	1 (0.3)

## Materials

### Simulated Teleconferencing Interaction Videos

**Fig. 1:**
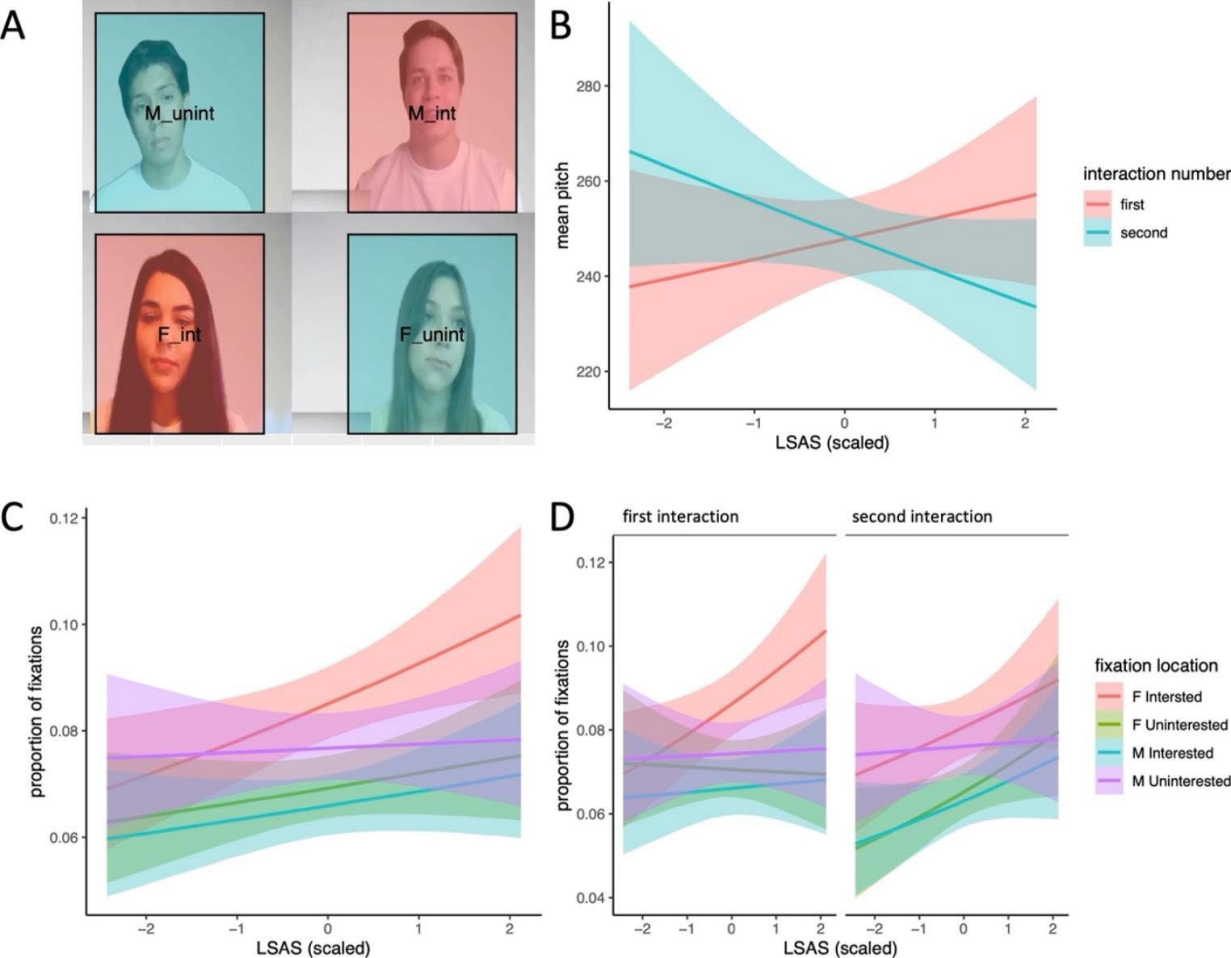
(A) Depicts a frame from the simulated teleconferencing interaction paradigm, with the regions of interest (ROIs) for eye tracking overlayed on the audience members. Blue highlights are the uninterested audience members and red are the interested audience members. (B) Depicts the interaction between vocal pitch and social anxiety symptoms for the first and second simulated teleconferencing interaction among female participants. (C) Depicts the association between symptoms of social anxiety and proportion of gaze to each of the audience members; (D) Depicts the association between symptoms of social anxiety and proportion of gaze to each of the audience members for the first and second simulated teleconferencing interaction *Note*. LSAS = Leibowitz Social Anxiety Scale

The simulated teleconferencing interaction videos consist of an audience of four individuals in 4-quadrants of the screen (like in a typical teleconferencing call) – see Fig. 1A. Audience members were coached to behave as either interested (nodding, smiling, etc.) or uninterested (looking away, crossing arms). There were four pre-recorded audience members – two who identified as male and two who identified as female. The simulated teleconferencing interaction videos are available on OSF https://osf.io/r3tnw/?view_only=36bade5e692548b7987f2c805b034e6f.

### Audio Data

The audio recording zone in Gorilla Experiment Builder allowed participants’ audio to be recorded using their microphone. This was used in the current study to record participants’ voices during the simulated teleconferencing interaction (participants still needed to manually enable the microphone). Participants’ audio data were saved as a separate mp3 file in the task’s Audio Recording Zone Metrics after each simulated teleconferencing interaction.

### Eye Tracking Data

Gorilla allows researchers to capture gaze data through the implementation of an Eye Tracking Zone. This zone uses Webgazer.js to detect participants’ faces, after which the participants’ gaze locations on the screen are inferred in real time using prediction models (Papoutsaki et al., 2016). Gaze detection performance is tested before each exposure trial using Gorilla’s calibration mode. Nine calibration points were used. The eye tracking zone were then validated internally by examining whether the gaze location to each calibration point was accurate and additional calibration was prompted if gaze was closer to different calibration point(s) than the intended point. After the calibration phase, participant gaze data was collected once the simulated teleconferencing interaction videos started. Gorilla provides both the raw x and y coordinates of participants’ gaze location as well as normalized x and y coordinates (0,1). Given the wide range of screen-sizes, the normalized coordinates were used for all analyses. The gaze data were not reliant on specific internet-speed or hardware requirements and were sampled at approximately 10 Hz, although this varied somewhat based on the participant’s computer.

## Data Preparation

### Audio Vocal Processing

The audio data were analyzed using the *soundgen* package (Anikin, 2019) in R to compute proportion of vocalizations (in order to confirm participant engagement) and extract vocal acoustic variables of interest. We used the default optimization. Based on previous research examining vocal acoustic indicators and social anxiety, we extracted the following variables: *speaking duration* (as a proportion of total time), *vocal pitch*, and *amplitude* (reflecting decibel level). We excluded participants who spoke for less than 10% of the allocated time available to them (n = 4, 1.5%), On average participants spoke for 72.75% (SD = 13.95%) of the available time.

### Eye Tracking Processing

Individual gaze datapoints were included if the ‘face confidence’ was greater than 0.5 (based on guidelines provided by Gorilla Experiment Builder) which reflects the face of the participant being accurately captured. Additionally, participants were excluded if more than 50% of their data was dropped (as a result of low ‘face confidence’). The data cleaning procedure led to the omission of 16 participants (6.0%). Another 14 participants (5.3%) were missing self-report or audio vocal data. Thus, the total sample that was available for eye tracking analyses was 236 participants.

Given that the eye tracking data used a prediction algorithm with variable sampling rates, true fixations cannot be reliably calculated. Instead, we computed the proportion of gaze points to each audience member ‘region of interest’ (ROI). ROIs were calculated manually using standardized quadrants to exclude gaze points at the border of each audience member which could not be reliably classified. This approach takes into account variability in sampling (i.e., number of gaze points) as well as the general noisiness of the web camera eye tracking. Gaze data were separately calculated when the participant was listening (the first half of the simulated teleconferencing interaction) and speaking (the second half of the simulated teleconferencing interaction). In the current analyses we chose to examine gaze data only from the speaking portion of the simulated teleconferencing interaction, given that these data correspond most closely to existing public-speaking paradigms.

### Data Analysis

Analyses were conducted using Bayesian regression with the *brms* package (Bürkner, 2017) in R. To address the multiple dependent variables across the SUDs and vocal acoustic data, we conducted multivariate, multilevel analyses with LSAS or PRCA as the predictors and individual participant as a random-intercept to account for variance across the two completed simulated teleconferencing interactions. Based on data showing sex differences in audio vocal features (Weeks et al., 2012), audio vocal analyses were conducted separately based on participants’ self-reported sex. Gaze analyses were conducted with proportion of gaze as the dependent variable, gaze location and LSAS or PRCA as predictors with a random-intercept for each individual participant. We retained each audience member as a unique region of interest (ROI) given the small number of ROIs and different non-overlapping audience member identities associated with each ROI (male/female; uninterested/interested).

Interaction order (simulated teleconferencing interaction 1 or simulated teleconferencing interaction 2) was included as a covariate in all analyses, and was also tested as a moderator for all analyses. Based on the recommendation of Banner et al. (2020) we used uninformative priors (with estimates centered on zero with reasonable values for the standard deviation) for the SUDs and vocal acoustic analyses. Informative priors were specified for the gaze analyses and bias was estimated by running the models with uninformative priors. Family link functions were specified based on reasonable assumptions about the distributions of the raw data and if necessary adjusted after plotting the posterior predictions against the distribution of the data (see R syntax for further details). We report results only for LSAS (symptoms of social anxiety) in the body of the manuscript; results related to PRCA (communication apprehension) were nearly identical and are made available in the supplementary materials. All data used in the analyses and the R syntax are available on OSF https://osf.io/r3tnw/?view_only=36bade5e692548b7987f2c805b034e6f.

## Results

### Validity of the Simulated Teleconferencing Interaction Task

The majority (71%) of participants stated that the interactions were realistic in an open-ended response to the question “Were the teleconferencing interactions realistic?” while 16% responded no and 11% did not respond. Responses varied somewhat given the open-ended nature of the question. One participant wrote “Very, at some points I forgot they were pre-recorded” while another response was more moderated “Kind of! They were more realistic than I had expected them to be, but still felt a bit stiff.”

Participants rated the uninterested audience members as less interested (M_male_uninerested_ = 3.73 (SD = 2.37), M_female_uninerested_ = 3.14 (SD = 2.37)) than the interested audience members( M_male_inerested_ = 7.35 (SD = 2.14), M_female_inerested_ = 6.71 (SD = 2.00)) and as eliciting a more negative emotional reaction (M_male_uninerested_ = 4.24 (SD = 1.92), M_female_uninerested_ = 3.73 (SD = 2.31)) than the interested audience members (M_male_inerested_ = 6.74 (SD = 2.37), M_female_inerested_ = 6.48 (SD = 2.00)).

### Subjective Fear and Associations with Symptoms of Social Anxiety

SUDs ratings were meaningfully greater than zero for anticipated fear *b* = 44.99, 95% HDI [42.02, 47.94], peak fear *b* = 46.34, 95% HDI [43.16, 49.54], and end fear *b* = 29.25, 95% HDI [26.12, 32.39]. There was a meaningful effect of interaction order on peak fear *b* =-13.80, 95% HDI [-16.64, -10.96] and end fear *b* = -5.30, 95% HDI [-8.40, -2.22], but not anticipated fear *b* = -1.93, 95% HDI [-5.21, 1.37].

As predicted, greater social anxiety symptom (LSAS) scores predicted greater anticipated fear *b* = 14.79, 95% HDI [12.32, 17.26], peak fear *b* = 12.89, 95% HDI [10.07, 15.75], and end fear *b* = 10.33, 95% HDI [7.46, 13.12]. Interaction order did not meaningfully moderate the relationship between social anxiety symptoms and anticipated fear *b* = 1.70, 95% HDI [-1.59, 4.95], peak fear *b* = 0.12, 95% HDI [-2.78, 3.01], or end fear *b* = -1.37, 95% HDI [-3.96, 1.17], indicating that the strength of the association was stable across both interactions.

### Audio Variables and Associations with Symptoms of Social Anxiety

For males, social anxiety symptoms were negatively associated with mean vocal amplitude *b* = -0.009, 95% HDI [-0.017, -0.0003], but not mean pitch *b* = -0.079, 95% HDI [-0.167, 0.008] or speaking duration *b* = 0.009, 95% HDI [-0.024, 0.044]; whereas female participants, displayed no meaningful associations between social anxiety symptoms and mean amplitude *b* = -0.001, 95% HDI [-0.007, 0.004], mean pitch *b* = -0.005, 95% HDI [-0.038, 0.028], or speaking duration *b* = -0.004, 95% HDI [-0.018, 0.009].

Moderating effects of social interaction order were observed only for female participants. Specifically, the order of social interaction moderated the effect of social anxiety symptoms on mean pitch *b* = -0.047, 95% HDI [-0.074, -0.019], where there was a positive association in the first interaction and a negative association in the second interaction (Fig. 1B), but not for mean amplitude *b* = 0.001, 95% HDI [-0.002, 0.003] or speaking duration *b* = 0.004, 95% HDI [-0.010, 0.019]. For male participants, interaction order did not moderate the effect of social anxiety symptoms on mean amplitude *b* = 0.002, 95% HDI [-0.002, 0.006], mean pitch *b* = 0.017, 95% HDI [-0.133, 0.099], or speaking duration *b* = -0.004, 95% HDI [-0.029, 0.020].

### Gaze Variables and Associations with Symptoms of Social Anxiety

There was a greater proportion of gaze to the interested female audience member compared to the uninterested female audience member *b* = -0.23, 95% HDI [-0.31, -0.14], uninterested male audience member *b* = -0.12, 95% HDI [-0.20, -0.03], and interested male audience member *b* = -0.28, 95% HDI [-0.37, -0.19]. There was no meaningful association between symptoms of social anxiety and overall proportion of gaze to audience members *b* = 0.05, 95% HDI [-0.11, 0.02].

Greater social anxiety symptoms were associated with a greater gaze to the interested female audience member compared to the uninterested male audience member *b* = -0.08, 95% HDI [-0.16, -0.01], but not compared to the uninterested female audience member *b* = -0.05, 95% HDI [-0.12, 0.03], or the interested male audience member *b* = -0.05, 95% HDI [-0.13, 0.03]. See Fig. 1C.

Order of interaction moderated the effect of social anxiety on gaze to the interested female audience member compared to the uninterested female audience member *b* = 0.14, 95% HDI [0.02, 0.26], such that there was relative avoidance of the uninterested female audience in the first interaction compared with the second. Interaction order did not moderate the effect of social anxiety on gaze to the interested female audience member compared to the uninterested male audience member *b* = 0.03, 95% HDI [-0.08, 0.15] or interested male audience member *b* = 0.09, 95% HDI [-0.03, 0.21]. See Fig. 1D.

## Discussion

This study introduces a new simulated teleconferencing interaction paradigm that can be used in remote research and has been made publicly available. In a relatively large sample, we found that most participants reported the interactions to be realistic. Additional support for this conclusion comes from participants’ reporting elevated social evaluative threat (ratings of anticipated, peak, and end SUDS). The strong associations between social evaluative threat and symptoms of social anxiety also suggests that responses to simulated teleconferencing interactions are likely to be valid for differentiating severity of social anxiety. Taken together, our findings suggest that this simulated teleconferencing interaction paradigm is an easily implemented remote paradigm that is effective for eliciting social evaluative threat.

Analyses examining mechanisms (vocal auditory and gaze related) were mostly consistent with prior findings. For example, our finding that greater symptoms of social anxiety were associated with greater pitch is similar to findings reported in prior published research (Galili et al., 2013; Weeks et al., 2012), although in our sample, this association was only present among female participants and only in the first interaction. Additionally, we found that greater social anxiety was associated with decreased vocal amplitude in male participants. It is also worth noting that a recent study (Alon-Ronen et al., 2022, n.p.) did not find an association between vocal pitch and symptoms of social anxiety in male or female participants. There is relatively little research on audio vocal indicators and social anxiety, making it an important area for future investigation. Research on audio vocal indicators of social anxiety have focused primarily on their utility as a prediction tool for augmenting self-report. Given that the primary focus of the current study was to report on the utility of our simulated teleconferencing interaction paradigm, we chose to examine only a small sample of possible audio vocal indicators grounded in prior research (Galili et al., 2013; Weeks et al., 2012). Exploring a wider range of audio vocal indicators would be worthwhile in future investigations.

We predicted that participants would display avoidance of uninterested (socially threatening) audience members regardless of gender. Contrary to expectation, avoidance of the uninterested female audience member was present only in the first interaction, but was no longer present in the second interaction, whereas avoidance of the uninterested male audience member compared to the interested female audience member was stable throughout both interactions. Additionally, there was no relative relationship between social anxiety and avoidance of uninterested audience members compared to the interested male audience member. Avoidance of uninterested audience members is consistent with prior research (Chen et al., 2015; Rubin et al., 2020), but the current findings lend an additional nuance with regards to *audience member* sex suggesting that future studies should consider *participant* sex, gender, race, ethnicity or other facets of identity and how they intersect with attentional processes during social stressors. In small samples such analyses can be challenging to conduct for several reasons; however, online recruitment offers the opportunity for more extensive recruitment of participants facilitating appropriately powered samples for addressing such questions.

Several limitations are important to note. First, our sample comprised mostly undergraduates, as were the actors in the simulated teleconferencing interaction videos. The aim of matching the participants with a specific relatively young demographic was to test this paradigm for individuals who would already be frequently engaging in these types of interactions. Yet, older adults may respond differently to the simulated teleconferencing interactions and thus replication with a more general non-student sample is needed. Second, some participants noted that the time given to respond to the question was too long. Future research may consider shortening the response period which could be easily accomplished by trimming the stimulus video length. Alternatively, participants could be given the option to end their response which would provide a measure of persistence. Third, the data on the qualitative reactions of participants to the simulated teleconferencing interactions were limited. Expanding the collection of qualitative data addressing participants’ reactions to this simulated teleconferencing interaction paradigm should be a high priority for future work in this area. Fourth, the validity the of the gaze data is difficult to ascertain without a method for external validation. Validation of this web camera eye tracking method using high resolution wearable eye trackers would offer an empirical solution to optimize data cleaning in future studies. While the audio vocal data appeared sound (pun intended!), customizing the optimization parameters is an important consideration that may influence the reliability of these findings. Additionally, identifying methods to collect remote physiological data synchronously with the simulated teleconferencing interactions would offer a potentially promising addition to subjective reports of distress. In future research it would be worth comparing the simulated teleconferencing interaction approach to a more standard public speaking approach. Yet, while the simulated teleconferencing interactions are meant to feel more interactive than a public speaking challenge with a pre-recorded audience, it is still the case that interactivity is relatively limited. Development of a fully interactive paradigm outside of virtual reality would be of significant potential value as they would offer substantially increased flexibility and adaptability in testing a wider range of social scenarios.

Despite these limitations, the study provides evidence supporting the remote administration of a simulated teleconferencing interaction paradigm for studying putative mechanisms governing social anxiety and its attenuation. Future research is needed to test whether this paradigm might represent a cost-effective alternative to therapist-delivered exposure therapy.

## Electronic Supplementary Material

Below is the link to the electronic supplementary material.

Supplementary Material 1

## References

[CR1] Alon-Ronen, O., Shrem, Y., Keshet, Y., & Gilboa-Schechtman, E. (2022). *The Vocal Signature of Social Anxiety: Exploration using Hypothesis-Testing and Machine-Learning Approaches* (arXiv:2207.08534). arXiv. 10.48550/arXiv.2207.08534

[CR2] Anikin A (2019). Soundgen: An open-source tool for synthesizing nonverbal vocalizations. Behavior Research Methods.

[CR3] Anwyl-Irvine AL, Massonnié J, Flitton A, Kirkham N, Evershed JK (2020). Gorilla in our midst: An online behavioral experiment builder. Behavior Research Methods.

[CR4] Arad G, Shamai-Leshem D, Bar-Haim Y (2021). Social Distancing during a COVID-19 Lockdown contributes to the maintenance of social anxiety: A natural experiment. Cognitive Therapy and Research.

[CR5] Bürkner PC (2017). Brms: An R Package for bayesian Multilevel Models using Stan. Journal of Statistical Software.

[CR6] Chen NTM, Thomas LM, Clarke PJF, Hickie IB, Guastella AJ (2015). Hyperscanning and avoidance in social anxiety disorder: The visual scanpath during public speaking. Psychiatry Research.

[CR7] Croft RJ, Gonsalvez CJ, Gander J, Lechem L, Barry RJ (2004). Differential relations between heart rate and skin conductance, and public speaking anxiety. Journal of Behavior Therapy and Experimental Psychiatry.

[CR8] Felnhofer A, Kothgassner OD, Hetterle T, Beutl L, Hlavacs H, Kryspin-Exner I (2014). Afraid to be there? Evaluating the Relation between Presence, Self-Reported anxiety, and Heart Rate in a virtual public speaking Task. Cyberpsychology Behavior and Social Networking.

[CR9] Galili L, Amir O, Gilboa-Schechtman E (2013). Acoustic Properties of Dominance and request utterances in social anxiety. Journal of Social and Clinical Psychology.

[CR10] Hindo CS, González-Prendes AA (2011). One-Session exposure treatment for social anxiety with specific fear of Public speaking. Research on Social Work Practice.

[CR11] Huneke, N., Rowlatt, H., Hyde, J., McEwan, A., Maryan, L., Baldwin, D., & Garner, M. (2021). *A Modified Trier Social Stress Test to Investigate Social Anxiety using Videoconferencing Software: A Proof-of-Concept study*. PsyArXiv. 10.31234/osf.io/q5rbj 10.1016/j.psychres.2022.114770 35961154

[CR12] Kessler RC, Chiu WT, Demler O, Walters EE (2005). Prevalence, severity, and Comorbidity of 12-Month DSM-IV Disorders in the National Comorbidity Survey Replication. Archives of General Psychiatry.

[CR13] Liebowitz MR (1987). Social phobia. Modern Problems of Pharmacopsychiatry.

[CR14] Lin M, Hofmann SG, Qian M, Kind S, Yu H (2016). Attention allocation in social anxiety during a speech. Cognition and Emotion.

[CR15] McCroskey JC, Beatty MJ, Kearney P, Plax TG (1985). The content validity of the PRCA-24 as a measure of communication apprehension across communication contexts. Communication Quarterly.

[CR16] Nauphal M, Swetlitz C, Smith L, Rosellini AJ (2021). A preliminary examination of the acceptability, feasibility, and effectiveness of a Telehealth cognitive-behavioral Therapy Group for social anxiety disorder. Cognitive and Behavioral Practice.

[CR17] Niles AN, Craske MG, Lieberman MD, Hur C (2015). Affect labeling enhances exposure effectiveness for public speaking anxiety. Behaviour Research and Therapy.

[CR18] Papoutsaki, A., Sangkloy, P., Laskey, J., Daskalova, N., Huang, J., & Hays, J. (2016). Webgazer: Scalable webcam eye tracking using user interactions. *Proceedings of the Twenty-Fifth International Joint Conference on Artificial Intelligence*, 3839–3845.

[CR19] Rapaport MH, Clary C, Fayyad R, Endicott J (2005). Quality-of-life impairment in depressive and anxiety Disorders. American Journal of Psychiatry.

[CR20] Reeves R, Elliott A, Curran D, Dyer K, Hanna D (2021). 360° video virtual reality exposure therapy for public speaking anxiety: A randomized controlled trial. Journal of Anxiety Disorders.

[CR21] Reichenberger, J., Pfaller, M., & Mühlberger, A. (2020). Gaze Behavior in Social Fear Conditioning: An Eye-Tracking Study in Virtual Reality. *Frontiers in Psychology*, *11*. https://www.frontiersin.org/articles/10.3389/fpsyg.2020.00035 10.3389/fpsyg.2020.00035 PMC6989556 32038441

[CR22] Rubin M, Minns S, Muller K, Tong MH, Hayhoe MM, Telch MJ (2020). Avoidance of social threat: Evidence from eye movements during a public speaking challenge using 360°- video. Behaviour Research and Therapy.

[CR23] Rubin, M., Muller, K., Hayhoe, M. M., & Telch, M. J. (2022). Attention guidance augmentation of virtual reality exposure therapy for social anxiety disorder: A pilot randomized controlled trial. *Cognitive Behaviour Therapy*, 1–17. 10.1080/16506073.2022.2053882. 10.1080/16506073.2022.2053882 PMC9458616 35383544

[CR24] Weeks JW, Lee CY, Reilly AR, Howell AN, France C, Kowalsky JM, Bush A (2012). The sound of Fear”: Assessing vocal fundamental frequency as a physiological indicator of social anxiety disorder. Journal of Anxiety Disorders.

[CR25] Wong QJJ, Rapee RM (2016). The aetiology and maintenance of social anxiety disorder: A synthesis of complimentary theoretical models and formulation of a new integrated model. Journal of Affective Disorders.

[CR26] Yuen EK, Goetter EM, Stasio MJ, Ash P, Mansour B, McNally E, Sanchez M, Hobar E, Forte S, Zulaica K, Watkins J (2019). A pilot of acceptance and commitment therapy for public speaking anxiety delivered with group videoconferencing and virtual reality exposure. Journal of Contextual Behavioral Science.

